# Very-Low-Dose Clozapine as Maintenance Treatment for Psychosis in a Patient With Dementia With Lewy Bodies: A Case Report

**DOI:** 10.7759/cureus.78226

**Published:** 2025-01-30

**Authors:** Diogo F Rodrigues, Filipe Azevedo, Mário Cunha, Joao Azenha, Leonor Brito-Santana

**Affiliations:** 1 Psychiatry, Unidade Local de Saúde de Lisboa Ocidental, Lisbon, PRT; 2 Psychiatry, Hospital de Egas Moniz, Unidade Local de Saúde de Lisboa Ocidental, Lisbon, PRT

**Keywords:** clozapine, dementia with lewy bodies, lbd, pimavanserine, psychosis

## Abstract

Dementia with Lewy bodies (DLB) is a neurodegenerative disorder characterized by cognitive decline, motor symptoms, and other neuropsychiatric manifestations, including visual hallucinations, delusions and disorganized thought processes that can configure a psychotic episode. Managing psychosis in DLB is challenging due to hypersensitivity to antipsychotics. This case report describes a 72-year-old female patient with DLB who presented with psychosis and Parkinsonian symptoms. Initial management with olanzapine (10 mg) was effective for psychosis but led to motor worsening. The inclusion of levodopa-carbidopa and reduction of olanzapine were not effective in the resolution of symptoms. Transition to clozapine performed and resulted in stabilization of psychotic symptoms and motor symptoms improvement. Initially, the patient presented with severe symptomatic hypotension, that required the tapering off to a very low dose (6.25mg) without severe adverse effects. This report highlights the utility of very-low dose clozapine in managing psychosis in DLB and discusses therapeutic challenges.

## Introduction

Dementia with Lewy bodies (DLB) is the second most common cause of neurodegenerative dementia after Alzheimer's disease, accounting for approximately 10-15% of cases [1]. Core clinical features include progressive cognitive decline, fluctuating attention and alertness, visual hallucinations, and tremor, rigidity, and bradykinesia (parkinsonism) [1]. Neuropsychiatric symptoms, particularly psychosis characterized by visual and auditory hallucinations and delusions, are prominent and often debilitating in DLB [2].

Management of psychosis in DLB is particularly challenging due to patients' hypersensitivity to antipsychotic medications, which can exacerbate motor symptoms, via reduction in the dopamine availability on nigrostriatal pathways, which are already impaired in DLB and can induce severe adverse reactions, including neuroleptic malignant syndrome [1]. Clozapine, an atypical antipsychotic has a very interesting binding profile that can be used on DLB, because of the lack of occupancy of D2 receptors in the brain, but with strong blockade of H1 histaminic, 5HT2C receptors that contribute to weight gain, potent antagonism of M1 muscarinic, H1, and α1-adrenergic receptors that can contribute to sedation, excessive salivation, and severe constipation and hypotension. Besides that, clozapine also has cardiac toxicity and risk of myocardiopathy. Clozapine has been considered a potential treatment option for psychosis in DLB due to its minimal impact on motor function [3]. However, its use necessitates careful monitoring for adverse effects with regular medical evaluations, blood samples and EKG. These difficulties limit its widespread application [4].

This case report explores the role of very low-dose clozapine in managing psychosis in a patient with DLB, providing insights into its efficacy and safety profile.

## Case presentation

A 70-year-old woman presenting with symptoms of depressed mood, mild working memory impairment, decreased initiative and interest in daily activities, middle insomnia, and cognitive anxiety symptoms, mainly consisting of worries and fears. She sought help from a private psychiatrist and was diagnosed with moderate depression, initially treated with escitalopram 10 mg, without complete resolution of the clinical picture. After 1.5 years, she maintains irregular private follow-up, without frank improvement in the clinical picture. The family reports that six months after the onset of the referred symptoms, bradykinesia, resting tremor, and gait difficulties emerged, worsening over the following three months. At age 72, the patient was referred by family members to the emergency department presenting with a two-week evolution clinical picture of persecutory delusions directed at family members, second-person auditory-verbal hallucinations with pejorative content, and intermittent visual hallucinations. She exhibited fluctuating attention, irritability, labile affect, and poor insight into her condition. Episodes of insomnia and disorganized thought processes, including derailments, were also reported. The patient was admitted to the psychiatric ward for the above reasons. Neurology consultation was requested. Blood and urine tests showed no significant alterations and ECG showed no changes. Mental state examination revealed a patient with sustained attention alterations, elicited speech, psychomotor agitation, tremor, and speech without dysarthria or other significant alterations. Thought processes showed periods of loose associations, persecutory delusions directed at family members, and delusions of ruin. Depressed mood, labile affect. Intermittent visual hallucinations and second-person auditory-verbal hallucinations. No death ideas or self-harm ideation. With insight into the symptoms. Neurological examination revealed symmetrical facial expression, cogwheel rigidity in the upper limbs, and a pronounced postural tremor in the right upper limb. A cranial CT scan showed no acute intracranial pathological findings; however, there were signs of moderate-to-severe leukoencephalopathy in the centrum semiovale and corona radiata, likely chronic and microangiopathic in origin. Based on clinical presentation, a multidisciplinary team comprising neurology and psychiatry specialists confirmed a diagnosis of DLB.

Initial management included donepezil for cognitive symptoms and olanzapine was titrated to 10 mg, once daily, with resolution of psychotic symptoms during the inpatient stay, in 10 days after admission. Escitalopram was reduced during hospitalization, without discontinuation effects. After discharge, the woman was referred to specialized Geriatric Psychiatry consultation in the Community Mental Health Team and Neurology consultation. Three weeks after discharge, there was maintained resolution of depressive symptoms, with worsening of motor symptoms (bradykinesia, tremor, and gait impairment), necessitating the introduction of levodopa/carbidopa (100 mg/25 mg, three times daily). There was a need to initially reduce olanzapine to 7.5 mg and later to 5 mg. The reduction in olanzapine caused a worsening of the patient's anxiety. Given the previous partial response to escitalopram, it was again titrated up to 10 mg. To reduce the functional impact of ongoing working memory complaints, neurology suggested the introduction of donepezil, titrated up to 10 mg, once daily. This led to partial motor improvement without recrudescence of psychosis. Anxiety slightly improved one month after the reintroduction of escitalopram.

Due to persistent motor symptoms, weight gain and other concerns over prolonged olanzapine use, a careful switch to clozapine was initiated. Clozapine was titrated to 25 mg but subsequently reduced to 12.5 mg after four weeks due to orthostatic hypotension, which required a further reduction in the following two weeks to 6.25 mg. At this very low dose, psychotic symptoms remained stable over six months, and no hematological complications were observed. Medical observation, regular blood monitoring, initially weekly, and after three months monthly, and regular EKG was conducted throughout treatment.

At the most recent psychiatric consultation, the patient was alert, calm, cooperative, and oriented in time, place, and person. Her speech showed no alterations in prosody or language. Thought processes were organized, with no delusions or hallucinations. The patient demonstrated insight into her condition, and her mood, although slightly dysthymic, was congruent with her affect.

**Figure 1:**
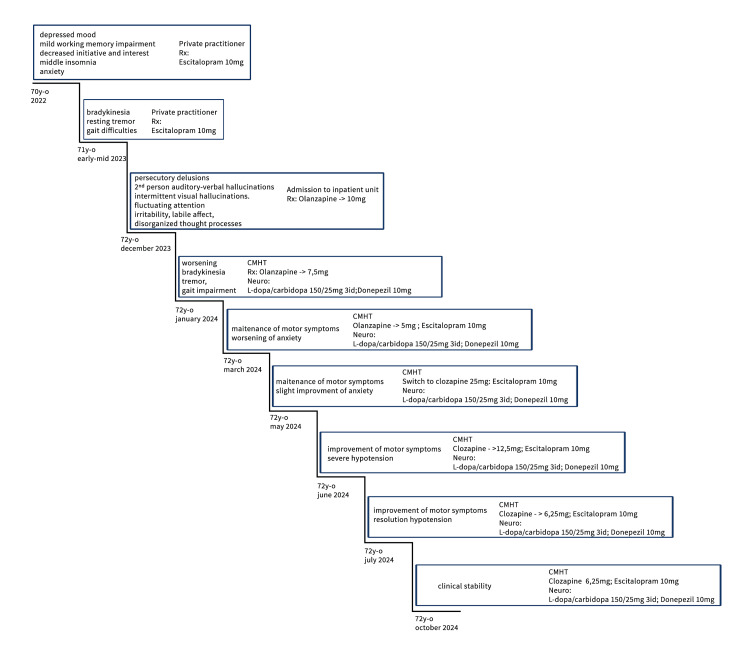
Timeline of clinical evolution CMHT: Community Mental Health Team

A more visual and simple understanding of the clinical evolution timeline is presented in Figure 1.

## Discussion

Managing psychosis in DLB presents significant therapeutic challenges due to the delicate balance required between alleviating neuropsychiatric symptoms and avoiding exacerbation of motor dysfunction. This case illustrates the potential efficacy and safety of low-dose clozapine in treating psychosis within this patient population. Clozapine’s unique pharmacological profile, characterized by the lack of occupancy of D2 receptors in the brain, but with strong blockade of H1 histaminic, 5HT2C receptors, potent antagonism of M1 muscarinic, H1, and α1-adrenergic receptors, renders it effective in mitigating psychotic symptoms with minimal impact on motor function symptoms. In this case, a very low dose of 6.25 mg daily was sufficient to maintain remission of psychotic symptoms over six months. For Parkinson's and DLB, clozapine usage can be considered low between 12.5 mg - 50 mg [3,5] with a mean of 25 mg of clozapine usage. Given the dosage used, we considered it to be a very low dose of clozapine, which shows the difference in our approach to this challenging clinical picture. Ballard et al. demonstrated that clozapine, at doses between 6.25 and 50 mg/day, is effective in controlling neuropsychiatric symptoms in DLB while avoiding significant motor side effects [3]. Additionally, Badwal et al. highlighted clozapine as one of the atypical antipsychotics with a relatively favorable safety profile, particularly when neuroleptic sensitivity is a concern in DLB [6]. Several concerns with clozapine therapy are pinpointed, such as the risk of agranulocytosis, necessitating regular hematological monitoring, constipation, hypotension and metabolic symptoms. In this patient, no hematological, cardiac or gastrointestinal complications were observed during treatment. Orthostatic hypotension was noted upon dose escalation but was manageable with the very low-dose adjustment. Badwal et al. emphasize the importance of integrating safety measures and close monitoring when prescribing clozapine for DLB psychosis, which aligns with the approach used in this case [6]. DLB patients often experience higher disease burden due to a combination of motor and neuropsychiatric symptoms. According to Badwal et al., psychosis significantly increases healthcare costs and caregiver burden in these patients [6]. Effective management strategies, such as those involving clozapine or other atypical antipsychotics, not only stabilize symptoms but may also delay institutionalization and improve quality of life. The concomitant use of levodopa/carbidopa, donepezil and escitalopram alongside clozapine contributed to improved motor function, anxiety symptoms and cognitive functionality without exacerbating psychosis in the three-month follow-up after the pharmacological adjustment. This synergistic approach highlights the importance of individualized treatment plans in DLB. As noted in the Portuguese Consensus (Monteiro et al., 2020), dopaminergic therapies are integral for motor symptom management, but their combination with antipsychotics must be carefully balanced to avoid destabilizing neuropsychiatric symptoms [7].

In addition to clozapine, recent advancements in the pharmacological management of DLB psychosis have introduced alternative therapies, such as pimavanserin [8-11]. This selective serotonin inverse agonist at 5-HT2A receptors offers a unique mechanism of action that avoids dopaminergic receptor binding, reducing the risk of motor side effects. Studies, including a recent systematic review [9] and case series [10,11], suggest that pimavanserine is effective in reducing hallucinations and delusions in DLB. However, its availability is limited in certain regions, including Portugal, and its long-term efficacy and safety require further investigation. Notably, combining pimavanserin with cholinesterase inhibitors may provide additional cognitive benefits, offering a promising avenue for integrated care strategies [12].

While pimavanserin presents a viable alternative for patients intolerant to clozapine, the two drugs’ comparative effectiveness in severe psychosis warrants further study. Current evidence suggests that clozapine may retain an advantage in managing more complex cases, particularly when psychotic symptoms are resistant to other interventions [5]. The absence of hematological monitoring requirements with pimavanserin represents an advantage, potentially increasing its accessibility and appeal in clinical practice. However, side effects such as sleep disturbances [9], prolongation of the QT interval [13]. Like other classes of antipsychotic drugs, there is an increased risk of death in elderly patients with dementia with pimavanserin [13].

Looking to the future, the integration of these therapies into personalized treatment frameworks will likely hinge on further advancements in precision medicine. Understanding patient-specific factors, such as genetic predispositions or receptor-binding profiles, may guide drug selection and improve outcomes. Additionally, research into novel therapeutic combinations and non-pharmacological interventions, such as cognitive-behavioral therapy or caregiver education, will be essential to addressing the multifaceted challenges of DLB.

## Conclusions

This case demonstrates the successful use of ultra-low-dose clozapine (6.25 mg) as maintenance therapy for psychosis in a patient with dementia with Lewy bodies. The treatment resulted in sustained remission of psychotic symptoms and stabilization of motor function over three months, without new significant adverse effects. These findings highlight the potential of clozapine as a viable option for managing psychosis in DLB, particularly when used at low doses and with careful monitoring, along with other pharmacological interventions used in Parkinson’s disease, like pimavanserine. However, further research is needed to confirm these outcomes in larger patient cohorts and to establish evidence-based guidelines for its use in this challenging population.
